# Fungal Biofilm Resistance

**DOI:** 10.1155/2012/528521

**Published:** 2012-02-08

**Authors:** Gordon Ramage, Ranjith Rajendran, Leighann Sherry, Craig Williams

**Affiliations:** ^1^Glasgow Dental School, School of Medicine, College of Medical, Veterinary and Life Sciences, University of Glasgow, Glasgow G2 3JZ, UK; ^2^Microbiology Department, Royal Hospital for Sick Children (Yorkhill Division), Dalnair Street, Glasgow G3 8SJ, UK

## Abstract

Fungal biofilm infections have become increasingly recognised as a significant clinical problem. One of the major reasons behind this is the impact that these have upon treatment, as antifungal therapy often fails and surgical intervention is required. This places a large financial burden on health care providers. This paper aims to illustrate the importance of fungal biofilms, particularly *Candida albicans*, and discusses some of the key fungal biofilm resistance mechanisms that include, extracellular matrix (ECM), efflux pump activity, persisters, cell density, overexpression of drug targets, stress responses, and the general physiology of the cell. The paper demonstrates the multifaceted nature of fungal biofilm resistance, which encompasses some of the newest data and ideas in the field.

## 1. Clinical Significance of Fungal Biofilms

Fungi represent a significant burden of infection to the hospital population. The use of broad-spectrum antibiotics, parenteral nutrition, indwelling catheters, or the presence of immunosuppression, or disruption of mucosal barriers due to surgery, chemotherapy, and radiotherapy are among the most important predisposing factors for invasive fungal infection [1]. *Candida* bloodstream infection is the third most common cause of nosocomial bacteremia in patients requiring intensive care and the most common etiologic agent of fungal-related biofilm infection. *C. albicans*, a normal commensal of human mucosal surfaces and opportunistic pathogen in immunocompromised patients, is most frequently associated with biofilm formation. Indwelling medical devices, such as intravascular catheters, can become colonized with *Candida *spp. allowing the development of adherent biofilm structures from which cells can then detach and cause an acute fungemia and/or disseminated infection. It has recently been shown that the cells that detach from the biofilm have a greater association with mortality than equivalent planktonic yeasts [2]. These implant-associated infections are inherently difficult to resolve and may require both long-term antifungal therapy and the physical removal of the implant to control the infection. Other non*albicans Candida* species associated with biofilm formation and catheter-related bloodstream or device-related infections include *C. glabrata*, *C. parapsilosis*, *C. dubliniensis*, *C. krusei*, and *C. tropicalis * [3–5]. 

Yeasts and filamentous fungi biofilm-related infections have also been increasingly described [6], including *Pneumocystis *[7], *Coccidioides* [8], *Aspergillus* [9], *Zygomycetes *[10], *Blastoschizomyces* [11], *Saccharomyces* [12], *Malassezia* [13], *Trichosporon* [14], and *Cryptococcus* [15]*. Cryptococcus neoformans* has been shown to colonize and subsequently form biofilms on ventricular shunts [15], peritoneal dialysis fistulas [16], prosthetic hip joints [17], and cardiac valves [18]. Different *Trichosporon *species can cause disseminated life-threatening infections associated with biofilm-related infections [14, 19, 20], including cardiac grafts [21], catheters [22], and breast implants [23]. *Malassezia pachydermatis* has been isolated from patients undergoing parenteral nutrition [13], *Blastoschizomyces capitatus* has been associated with catheter-related fungemia [11], *Saccharomyces cerevisiae *has been detected from dentures of stomatitis patients [24], and recurrent meningitis has been associated with a *Coccidioides immitis* biofilm at the tip of a ventriculoperitoneal shunt tubing [8]. 

There are also growing reports of the filamentous mould *Aspergillus fumigatus* being involved in biofilm infections. For example, in the respiratory tract, it can cause an aspergilloma, which is a localised infection consisting of a spherical mass of hyphae. Aspergillary bronchitis has also been reported, which is characterized by bronchial casts containing mucus and mycelia [25]. Bronchopulmonary lavage (BAL) of patients with aspergillosis may also reveal the presence of numerous hyphae in the form of a complex multicellular mycetoma structure samples when examined histologically [26]. In addition to this, it has been reported to cause serious biomaterial-related infections of joint replacements, catheters, heart valves, cardiac pace makers, and breast augmentation implants [27–30]. The urinary tract, whilst less frequently associated with *A. fumigatus*, has been reported to support an aspergilloma [31, 32]. It is also frequently associated with complex sinus infections, which in canines have been described as superficial mucosal fungal plaque [33–36]. 

It is increasingly clear that a diverse panel of fungi have the capacity to form biofilms, and as such our knowledge of fungal biofilms has improved dramatically. Through work primarily with *C. albicans*, we now have a clearer perspective on the molecular characteristics of fungal biofilm development [3, 6, 37, 38]. Clinically, these are important as they are refractory to antifungal treatment, which poses a major problem to clinicians as the dose required to eradicate the biofilm can exceed the highest therapeutically attainable concentrations of antibiotics [39]. The focus of this paper is to provide an up-to-date understating of the key factors responsible for the failure of antifungal agents against fungal biofilms.

## 2. Biofilm Basics

Microbiologists have historically studied planktonic (free floating and homogeneous cells) in pure culture. However, there has been a paradigm shift as the link between sessile (surface attached and heterogeneous cells) and microbial pathogenesis and human infection is now widely accepted [40]. It is apparent that a wide range of bacteria and fungi are able to alternate between planktonic growth and sessile multicellular communities, commonly referred to as biofilms. Estimates suggest that up to 80% of all microorganisms in the environment exist in biofilm communities [41]. 

Biofilms are defined as highly structured communities of microorganisms that are either surface associated or attached to one another and are enclosed within a self-produced protective extracellular matrix (ECM) [6]. The advantages to an organism of forming a biofilm include protection from the environment, resistance of physical and chemical stress, metabolic cooperation, and a community-based regulation of gene expression. In recent years, there has been an increased appreciation of the role that fungal biofilms play in human disease as microbes growing within biofilms exhibit unique phenotypic characteristics compared to their planktonic counterpart cells, particularly increased resistance to antimicrobial agents [6]. In addition to providing safe sanctuary for microorganisms, biofilms may also act as reservoirs for persistent sources of infection in a patient and as such adversely effect the health of an increasing number of individuals, including patients with HIV-infection, cancer, transplants, patients requiring surgery or intensive care, and newborn infants [3, 42].

The adhesion and colonization of complex fungal populations onto biological and innate surfaces, such as the oral mucosa or denture acrylic substrates, is commonplace for clinically relevant fungi [43–46]. A wide variety of environmental factors contribute to the initial surface attachment, including the flow of the surrounding medium (urine, blood, saliva, and mucus), pH, temperature, osmolarity, bacteria, presence of antimicrobial agents, and host immune factors [47–52]. Fungal biofilms have defined phases of development that have been described through the use of defined model systems [51, 53–58]. These key phases include arriving at an appropriate substratum, adhesion, colonisation, extracellular matrix (ECM) production, biofilm maturation, and dispersal [37, 55, 59]. Understanding this entire process has enabled us to begin to unravel how some of the mechanisms are involved in resistance.

## 3. Studying Fungal Biofilm Resistance

Initial studies that began to investigate biofilm resistance were basic, investigating antifungal effects purely at the phenotypic level through descriptive analyses. The pioneering work by Julia Douglas's group working on *C. albicans *biofilms utilised some of the earliest models, from which quantitative assessment using dry weight measurements, tetrazolium salt (MTT) reduction assays, and incorporation of [^3^H] leucine were described [60, 61]. These simple static models were expanded to include flow, which was shown to alter antifungal susceptibility [47]. However, typically these models were cumbersome, requiring expert handling, longer processing times, and the use of specialized equipment not generally available. Therefore, methods for rapid high-throughput testing were preferable, and around this time, the 2,3-bis(2-methoxy-4-nitro-5-sulfophenyl)-5-[(phenylamino)-carbonyl]-2H-tetrazolium hydroxide (XTT) colorimetric method was described for investigating yeast adhesion and susceptibility [62, 63]. This assay measures the collective metabolic activity of the cells within biofilm and is used as the basis for developing a standardized high-throughput susceptibility screen on *Candida* biofilms, which is now widely used by the research community [55, 58]. The XTT assay is noninvasive and nondestructive, requiring minimal postprocessing of samples compared to other methods, such as viable cell counts [64]. Using this technique, multiple microtiter plates can be processed simultaneously without compromising accuracy. A caveat to its use is that it does not quantify biofilm-dependant characteristics, such as biomass or morphological status, and caution must be exercised when evaluating XTT data from different isolates as there is often dramatic variability between strains [65]. Therefore, it should only be used for direct comparison of a treated isolate to an untreated control rather than absolute quantification of biofilm formation *per se*. The next breakthrough in high-throughput biofilm testing has recently been described where nanoproduction of *C. albicans *biofilms is achievable, creating 768 equivalent and spatially distinct nanobiofilms on a single glass microarray [66]. However, it remains to be determined whether there is an assay system sensitive enough to quantify the metabolic activity of each nanobiofilm. 

The recent interest in *Aspergillus *and *Cryptococcus *biofilms, as well as others like *Pneumocystis*, has led to further modifications of these, but in essence the platforms and techniques remain largely similar [67]. For example, Martinez and Casadevall (2006) developed a microtiter plate biofilm assay for *C. neoformans* to determine the susceptibility profiles of *in vitro* sessile structures [68]. Similarly, a 96-well-based biofilm model for *A. fumigatus* has been described and used to determine the susceptibility profiles and resistance mechanisms of conidia and adherent hyphal biofilms using an XTT-based reduction and Alamar blue assays [69–71]. The oxidation reduction indicator Alamar blue has also been shown to be a reproducible and cheaper alternative to XTT in recent years, which merits further study [72, 73].

As mentioned, the presence of flowing liquid over the biofilm can alter antifungal sensitivity [47]. There is a growing range of flow systems utilized to model biofilm development [51, 56, 74–76]. For example, a “seed and feed” modified Robin's device that permits multiple biofilms to be formed under constant flow conditions, cylindrical cellulose filters, constant depth film fermenters, perfusion fermenters, flow chambers, and a Robbin's device have all been described [51, 52, 54, 55, 77, 78]. The Lopez-Ribot group recently described a simple flow model based on a gravity-fed flow method that enabled the group to demonstrate that biofilms were thicker and more resistant to polyenes and echinocandins by 4- and 2-fold, respectively [79]. Interestingly, perfusion of biofilms created under flow with these two antifungal agents showed time- and dose-dependant activity, which were potent against dispersed cells [80]. These systems will prove useful for future investigations of invasive candidiasis where biofilms are common, particularly for catheter-related infections in the ICU, where there is a growing interest in catheter-lock therapy [81, 82]. In addition, there are now also a significant number of biofilm models available for *in vivo *investigations, and many of these have been utilised to elucidate biofilm resistance mechanisms [83], including an implanted chamber under the skin [71], catheter models [84, 85], vaginal model [86], and denture model [87].

## 4. Fungal Biofilm Resistance Mechanisms

One of the defining characteristics of biofilms is their increased resistance to antimicrobial agents. Fungi have been reported to be up to 1000-fold more resistant to antifungal agents than planktonic free-floating cells, yet this recalcitrance to antimicrobial therapy has yet to be fully elucidated [14, 58, 88]. Despite some antifungal agents being efficacious against fungal biofilms, particularly the echinocandins and liposomal amphotericin B formulations, the intrinsic resistance exhibited by these complex structures has promoted detailed investigation [70, 89–92]. 

**Figure 1:**
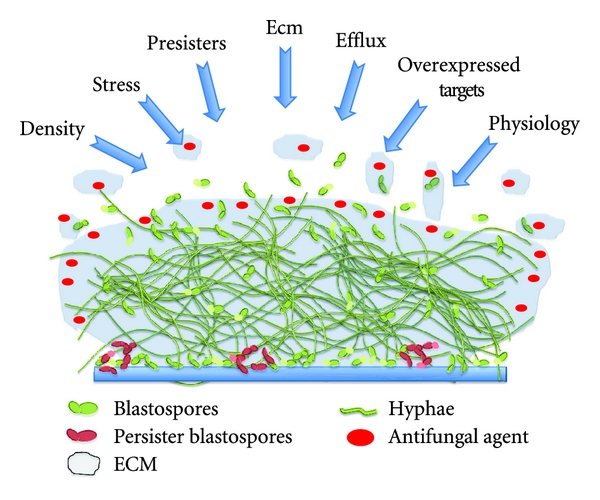
Schematic overview of fungal biofilm resistance mechanisms. Generic overview of key biofilm resistance mechanisms associated with *C. albicans*, but which are likely to be common to other fungi. This figure illustrates the density and complexity of the *C. albicans *biofilm, with different morphotypes present surrounded by ECM. The arrows represent the different factors that drive antifungal resistance within the biofilm, including density, stress, persisters, ECM, efflux, overexpressed targets, and the general physiology of the biofilm. These have been placed according to their contribution to resistance, with those that have a greater effect closer to the middle and those with less impact at the edges.

**Figure 2:**
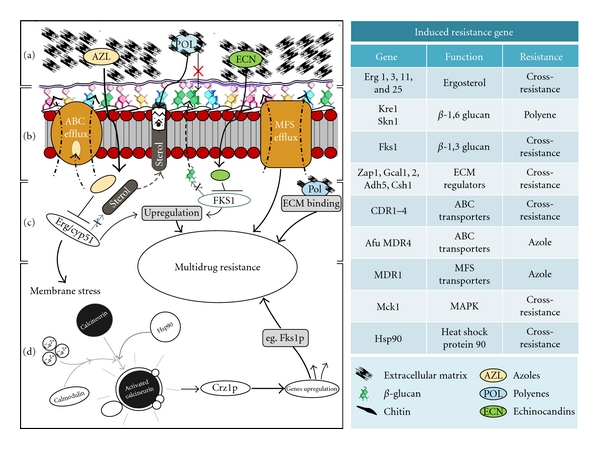
Molecular mechanisms of fungal biofilm resistance. Antifungal drug resistance in fungal biofilms is both complex and multifactorial. The diagram illustrates the mechanisms of different class of antifungal agent action (azoles [AZL], polyenes [POL], and echinocandins [ECN]) and resistance: (a) the layer of ECM present in the biofilm shields the cells from antifungal agents by binding and reduced penetration; (b) the membrane transporter system ABC and MFS efflux pumps extrude antifungal molecules and reduce the intracellular concentration; (c) mutation in *ERG*, *Cyp51,* and *FKS1* genes alters the drug target leading to cross-resistance; (d) antifungal pressure induces stress responses, such as the calcineurin signalling pathway, which is activated, and coping responses occur through upregulation of various signal transducers. On the right hand side, the table lists different resistance genes and their functions, and antifungal agents affected.

Antifungal resistance is both complex and multifaceted. It can be inducible in response to a compound, or an irreversible genetic change resulting from prolonged exposure. Specifically, these include alterations or overexpression of target molecules, active extrusion through efflux pumps, limited diffusion, tolerance, and cell density, which are all characterised mechanisms utilised by fungi to combat the effects of antifungal treatment [93]. Planktonic cells generally rely on irreversible genetic changes to maintain a resistant phenotype, whereas biofilms are able to persist due to their physical presence and the density of the population, which provides an almost inducible resistant phenotype irrespective of defined genetic alterations. The following section will now describe some of the pivotal factors that play a role in fungal biofilm resistance, which are summarised in Figures 1 and 2. 

### 4.1. Physiological State

The general physiological state of cells in sessile populations has also been implicated to influence the susceptibility profiles of biofilms. Metabolic dyes assays (e.g., XTT-based assays) confirm that cells within biofilms are undergoing mitochondrial respiration during development [55, 58, 62, 65, 70]. Other factors including the effect of growth rate on *C. albicans* biofilm resistance have also been studied, where varying the rates was shown to play no role in resistance to amphotericin B [54]. Similarly, biofilms of *C. albicans* grown under glucose- and iron-limited conditions were shown to both be highly resistant to amphotericin B [94]. Furthermore, studies of biofilms grown under anaerobic conditions demonstrated that *C. albicans *biofilms were resistant to the high levels of amphotericin B and different azole antifungals [95]. Nevertheless, factors including pH, temperature, oxygen availability, and other environmental stresses will alter the biofilm architecture, and possibly antifungal sensitivity [96, 97]. Therefore, whilst the physiological state of the cell may have a minor role in resistance (e.g., dormancy), it is more likely that more complex factors are involved. 

### 4.2. Cell Density

The architecture of biofilms is highly ordered to enable the perfusion of nutrients and expulsion of waste products. Mature biofilms, whilst densely populated, exhibit spatial heterogeneity with microcolonies and water channels being present, and features are common to both bacterial and fungal biofilms [55, 98, 99]. Cell density is therefore an important resistance factor within complex biofilm populations of yeast and filamentous fungal biofilms, particularly towards azoles. It was demonstrated that both planktonic and resuspended biofilm cells exhibited azole sensitivity at low cell numbers (10^3^ cells/ml), which became increasingly resistant as the density of the cells increased tenfold [100], a phenomenon also been demonstrated in *A. fumigatus* [101]. Both our group and others have shown phase-dependant increased antifungal resistance in *A. fumigatus *and *C. albicans*, respectively [70, 102], which support the idea that the physical density of the cells within the biofilm produces recalcitrance to antifungal agents. 

Within dense biofilms, there is cooperation between individual cells through quorum sensing processes, which provides the ability of microorganisms to communicate and coordinate their behaviour via the secretion of signalling molecules in a population-dependent manner [103]. In fungi, this was first described in *C. albicans* when Hornby and colleagues identified farnesol *trans*, *trans*-3,7,11-trimethyl-2,6,10-dodecatrien-1-ol [104]. Exposing *C. albicans* to exogenous farnesol results in genome wide responses, including activation of genes involved in drug resistance (Ca*FCR1 *and Ca*PDR16*) [105, 106]. It has now been shown that quorum sensing in *C. albicans *is likely driven by the two-component regulatory system of Chk1p [107]. However, when deleted the *Δchk1* strain shows a similar azole resistance profile to that of wild type [100], indicating that the regulatory circuit controlling biofilm resistance may be yet to be discovered, or cell density is not a defined biofilm resistance factor. However, given that echinocandins are highly effective against biofilms suggests cell density has a limited effect against this compound [92]. In addition, previous work has shown that disrupted biofilms that are resuspended and tested using the CLSI methodology in comparison to planktonic cells retain a resistant phenotype [71, 108], indicating alternative mechanisms of resistance. 

### 4.3. Overexpression of Drug Targets

The azoles are generally fungistatic against yeasts, including *Candida *species, and fungicidal against moulds, such as *Aspergillus *species. The fungistatic nature of the azoles towards *C. albicans* induces a strong directional selection on the surviving population to evolve drug resistance [109, 110]. In fact, high levels of azole resistance in *C. albicans *clinical isolates often accumulate through multiple mechanisms including the alteration of Erg11 [109]. Azoles actively target the 14*α*-demethylase enzyme encoded by ERG11, blocking ergosterol biosynthesis and leads to depletion of the ergosterol content of membranes and results in the accumulation of toxic sterol pathway intermediates, such as 14 *α*-methylergosta-8,24(28)-dien-3b,6a-diol, which inhibits growth [111, 112]. The principle drug target, Erg11p, can develop point mutations or be overexpressed [111–113]. Common mutations in the Erg11p that confer moderate azole resistance are S405F, Y132H, R467K, and G464S [114–116]. 

Given the importance of ergosterol as a target of azoles and the high level resistance exhibited by these structures, then the sterol composition of *C. albicans* biofilms has been investigated. Sterol analyses showed that ergosterol levels were significantly decreased in intermediate (12 h) and mature phases (48 h), compared to those in early-phase biofilms (6 h) [102]. In contrast, in one of the first *C. albicans *biofilm studies to use microarray analysis, overexpression of Ca*ERG25* and Ca*ERG11 *was reported [56]. Alteration of ergosterols in biofilm membranes may explain their resistance to both azole and polyene-derived antifungal agents. For example, *C. albicans* biofilms cultured in a flow cell for 36 h were compared to planktonic cells, where it was shown that a subpopulation of blastospores from the biofilm were 10 times more resistant to amphotericin B than planktonic populations [76]. Transcriptional analysis of this biofilm subpopulation for genes from the beta-1,6-glucan pathways indicated a possible association between the high level of resistance and upregulation of Ca*SKN1*, and Ca*KRE1* in the biofilm blastospore population compared to exponential and stationary phase planktonic *C. albicans *cells. Therefore, changes in both the cell membrane and the cell wall may be important determinants of resistance in the biofilm. Subsequent work in *C. albicans *has shown that transcriptional responses in young and mature biofilms after exposure to high doses of fluconazole or amphotericin B demonstrated differential antifungal drug responses [117]. Exposure of both young and mature biofilms to fluconazole induced upregulation of genes encoding enzymes involved in ergosterol biosynthesis (Ca*ERG1*, Ca*ERG3*, Ca*ERG11,* and Ca*ERG25*), particularly biofilms exposed for longer periods (22 h), whereas treatment of both young and mature biofilms with amphotericin B resulted in an overexpression of predominantly Ca*SKN1*, with a modest upregulation of Ca*KRE1*. Removal of the antifungal in this study depleted further transcriptional changes, except for Ca*SKN1, *which was impacted by prior fluconazole exposure. It was speculated that this is related to biofilm regrowth. Increased ergosterol genes have also been reported *in vivo *in a *C. albicans *central venous catheter biofilm model, demonstrating the importance of the molecule within the biofilm [118]. 

Induction of ergosterol genes has also been described in *C. dubliniensis*, where incubation with fluconazole and formation of biofilm was coupled with upregulation of the Cd*ERG3* and Cd*ERG25* [119]. Moreover, upregulation of genes involved with ergosterol biosynthesis has been described in *C. parapsilosis* biofilms [120], which are also resistant to azole antifungal therapy [121]. Overall, these data highlight the importance of ergosterol in biofilm resistance, particularly with respect to azoles, which indirectly inhibit their biosynthesis. Recent studies have shown that simvastatin, which impairs cholesterol metabolism in humans, is capable of inhibiting *C. albicans *biofilms, thus providing a potential novel strategy of combating these tenacious infections [122]. 

### 4.4. Efflux-Pump-Mediated Resistance

The primary molecular mechanism leading to high-level azole resistance in *C. albicans,* that is, increased efflux of drug mediated mostly by the ATP-binding cassette (ABC) and the major facilitator superfamily (MFS) transporters [123–125]. The ABC transporters in *C. albicans* constitute a multigene family, which includes several CDR genes (CDR1-4) [126, 127]. The ABC transporters include a membrane pore composed of transmembrane segments and two ABCs on the cytosolic side of the membrane, which provide the energy source for the pump [128, 129]. Importantly, multiple antifungal agents can be substrates for these transporters, and thus, their overexpression can lead to cross-resistance among different drugs, particularly azoles. Among members of the MFS, which are secondary transporters and use proton-motive force across the plasma membrane, the MDR1 gene encodes a major facilitator that has been implicated in *C. albicans* azole resistance, and its overexpression leads to exclusive fluconazole resistance [46, 113]. Echinocandin sensitivity is unaffected by efflux pumps [130]. 

Genes encoding for drug efflux pumps have been reported in biofilms to be differentially regulated during development and upon exposure to antimicrobial agents include Ca*CDR1, CaCDR2, *and Ca*MDR1* [102, 108, 131, 132]. In the first study to investigate the role of efflux pumps, it was demonstrated that expression of genes encoding both types of efflux pump were upregulated during the course of biofilm formation and development. Both Ca*CDR1* and Ca*CDR2* were upregulated in 24 and 48 h biofilms, whereas Ca*MDR1 *was transiently upregulated at 24 h [108]. However, their contribution to resistance in the biofilm phenotype was placed in doubt when a set of *C. albicans *isogenic strains deficient in efflux pumps carrying single- and double-deletion mutations (*Δcdr1*, *Δcdr2*, *Δmdr1*, *Δcdr1/Δcdr2*, and *Δmdr/Δcdr1*) that rendered the cells hypersusceptible to fluconazole when planktonic retained the resistant phenotype during biofilm growth. In a subsequent investigation, *C. albicans* biofilms were formed through three distinct developmental phases that were associated with high fluconazole resistance. Again, the same set of isogenic *C. albicans* strains were utilised where it was shown that 6 h old biofilms formed by double and triple mutants were >4- to 16-fold more susceptible to fluconazole than the wild-type strain [102]. At 12 and 48 h, all the strains became highly resistant to this azole, indicating lack of involvement of efflux pumps in resistance at late stages of biofilm formation. In cell density studies of the efflux pump isogenic strains, these remain hypersensitive at low cell concentrations yet resistant at high cell concentrations and in biofilm, indicating a contributory resistance role of cell density [100]. Nevertheless, similar to the study by Ramage and coworkers [108], *C. albicans* biofilms were shown to express Ca*CDR* and Ca*MDR1* genes in all three phases (6, 12, and 48 h), whilst planktonic cells expressed these genes transiently. In fact, GFP promoter studies have shown induction of efflux pumps after 15 min adherence to provide a tolerant biofilm phenotype [132]. Animal studies have also shown that biofilms formed on implanted catheters express efflux pumps [84, 118]. Transcript upregulation of Ca*CDR2* at 12 h (1.5-fold) and Ca*MDR1* at both 12 h (2.1-fold) and 24 h (1.9-fold) was demonstrated [118]. In *C. glabrata, *similar results are reported, where expression of Cg*CDR1* and Cg*CDR2* was investigated during the early (6 h), intermediate (15 h), and mature (48 h) phases of biofilm development. At 6 h and 15 h, the biofilms exhibited approximately 1.5- and 3.3-fold upregulation of Cg*CDR1* and 0.5- and 3.1-fold upregulation of Cg*CDR2*, respectively, in comparison to planktonic cells [133]. Expression of Ct*MDR *in *C. tropicalis *biofilms has also been reported [134]. 

Collectively, these studies suggest that multifaceted, phase-specific mechanisms are functional in resistance of fungal biofilms. This is confirmed in studies of *A. fumigatus *biofilm resistance. Mutations within the *cyp*51A gene, which alters the ergosterol biosynthesis pathway, have been reported to cause azole resistance in *A. fumigatus * [135–138]. However, a recent study reported that 43% of azole-resistant isolates did not carry the cyp51A mutation, indicating that other mechanisms of resistance were responsible [139]. It was hypothesised that efflux-mediated mechanisms of resistance may explain this clinical resistance, which may also be important in biofilms. Sequence analysis suggests that *A. fumigatus *has 278 different MFS and 49 ABC transporters [140]. *A. fumigatus MDR *pumps have previously been shown to be associated with increased resistance to itraconazole [141, 142]. Currently, however, there is little evidence to suggest that these play an active role in clinical resistance [143]. Phase-specific analysis of resistance in *A. fumigatus *biofilms revealed increased resistance to azoles, polyenes, and echinocandins as each biofilm matured from 8 to 12 to 24 h [70, 71]. Biochemical analysis of efflux pump activity showed a significant increase in efflux pump activity in the 12 h and 24 h phases, and upregulation in 8 h germlings when treated with voriconazole. Moreover, inhibition of efflux pump activity with the competitive substrate (MC-207, 110) reduced the susceptibility to voriconazole by 5-fold. Quantitative expression analysis of Afu*MDR4* mRNA transcripts revealed a biphasic increase as the mycelial complexity increased (maximal at 12 h), which was coincidental with strain-dependant increase in azole resistance. Similar biphasic increases in *C. glabrata *CDR genes were also observed [133]. Voriconazole also significantly induced Afu*MDR4 *expression, which was also detected *in vivo* [71]. Global transcriptional analyses of voriconazole-treated *A. fumigatus *mycelia of over 2000 genes were shown to be differentially expressed, and amongst these was a cluster of 15 different transporters mRNA at significantly increased levels, including MDR proteins of the ABC and MFS classes, such as AfMDR1 and AfMDR2 [144]. Also, unpublished microarray studies of the different phases of *A. fumigatus *biofilms from our group revealed a cluster of pumps and transporters, which is similar to studies of *C. albicans * [131]. 

Collectively, this data in addition to the available literature support the hypothesis that efflux pumps are an important, but not exclusive, determinant of fungal biofilm resistance to azoles [143, 145]. Their primary role may be for homeostasis within complex environments to protect themselves from acute toxicity [146], but within clinical environments exposure to azoles dugs may enhance the levels of efflux pump expression, therefore either contributing towards or inducing clinical resistance [139]. However, it is likely that they play a greater protective (resistance) role in the early phases of biofilm growth until the production of ECM, one of the primary mechanisms of biofilm resistance.

### 4.5. Extracellular Matrix

ECM is a defining characteristic of fungal biofilms, providing the cells protection from hostile factors such as host immunity and antifungal agents [6]. In some of the pioneering works by the Douglas group, *C. albicans *ECM was shown to increase when biofilms are grown under dynamic flow conditions [47, 48, 53]. However, subsequent work has shown that while diffusion is hampered by ECM, penetration of antifungal drugs is not thought to play a key role in biofilm resistance [53]. Recent studies have provided new insights that suggest the chemical composition of ECM and its regulation may play a central role in resistance.

The composition of the ECM of these biofilms in *C. albicans* and *C. tropicalis* is complex, comprising of protein, hexosamine, phosphorus, uronic acid, and carbohydrates [147]. Recently, it has been shown that extracellular DNA is another important component of the ECM in C. *albicans* [148], as the addition of DNase improves the efficacy of polyenes and echinocandins, but not to azoles [149]. One of the principle carbohydrate components is beta-1,3 glucans, as treatment of *C. albicans *biofilms with beta-1,3 glucanase helps detach biofilms from a substrate [147]. Its contribution is confirmed in a series of investigations by the Andes group where it was shown to increase in *C. albicans* biofilm cell walls compared to planktonic organisms and was also detected in the surrounding biofilm milieu and as part of the ECM [150]. Beta-1,3 glucans have also been shown to increase in investigations of three specific phases of biofilm development grown on both denture acrylic and catheter substrates [151]. Its contribution to resistance was realized when it was also shown that biofilm cells walls bound 4- to 5-fold more azole than equivalent planktonic cells, and culture supernatant bound a quantifiable amount of this antifungal agent. Moreover, beta-1,3 glucanase markedly improved the activity of both fluconazole and amphotericin B. Addition of exogenous biofilm ECM and commercial beta-1,3 glucan also reduced the activity of fluconazole against planktonic *C. albicans in vitro* [150]. The group has recently shown that the ECM *β*-1,3 glucan is synthesised from Fks1p using a defined knockout and overexpressing strain [152]. This study demonstrated that beta-1,3, glucan is responsible for sequestering azoles, acting as a “drug sponge” and conferring resistance on *C. albicans *biofilms [152]. Further studies have shown that they are also responsible for sequestering echinocandins, pyrimidines, and polyenes [153]. This has been confirmed independently where AMB was shown to physically bind *C. albicans* biofilms and beta-glucans [154]. Subsequent studies have identified a role for the Ca*SMI1*, a gene involved in cell wall glucans, in biofilm ECM production and development of a drug-resistant phenotype, which appears to act through transcription factor Ca*Rlmp* and glucan synthase Fks1p [155]. 

In addition to Ca*FKS1, *a zinc-response transcription factor Ca*ZAP1* has been shown to be a negative regulator of ECM soluble beta-1,3 glucan in both *in vitro* and *in vivo C. albicans *biofilm models through expression profiling and full genome chromatin immunoprecipitation [156]. Conversely, two glucoamylases, Ca*GCA1* and Ca*GCA2*, are thought to have positive roles in matrix production. A group of alcohol dehydrogenases Ca*ADH5*, Ca*CSH1*, and Ca*LFD6* also have roles in matrix production, with Ca*ADH5* acting positively, and Ca*CSH1* and Ca*LFD6* acting negatively [156]. It is thought that these alcohol dehydrogenases generate quorum-sensing aryl and acyl alcohols, which coordinate biofilm maturation. Collectively, it appears that *C. albicans *ECM production is highly regulated and is a key resistance factor. It is also present on a number of other *Candida *spp., including *C. glabrata*, *C. parapsilosis*, *C. tropicalis, *and *C. dubliniensis * [157, 158]*.*

Significantly less is known regarding the role of *A. fumigatus* biofilm ECM in antifungal resistance. In an aerial static model, the presence of extracellular hydrophobic ECM is composed of galactomannan, alpha-1,3 glucans, monosaccharides, polyols, melanin, and proteins, including major antigens and hydrophobins [159]. This study demonstrated that hydrophobic matrix cohesively bound hyphae together, and that the matrix increased with maturity of the developing structure. Further studies report that a new galactosaminogalactan and the galactomannan were the major polysaccharides of the *in vivo A. fumigatus* ECM [160]. Extensive ECM production was also reported as the maturity of the biofilm increases [9]. For *A. niger, *after germination upon a support, the new hyphae also produce an ECM [161]. The production of ECM has also been reported elsewhere, where it has been shown to be produced on both polystyrene and cystic fibrosis (CF) bronchial epithelial cells by *A. fumigatus*, that were resistant to antifungal agents [162]. 

Martinez and Casadevall (2006) reported that *C. neoformans* also have the ability to form biofilm structures *in vitro* and produce ECM [68]. In *Pneumocystis *spp., confocal microscopy has revealed organisms enmeshed in ECM. Intense monoclonal antibody staining to the major surface glycoproteins and an increase in (1,3)-beta-D-glucan content were also evident, suggesting that these components contributed to resistance [7]. *Blastoschizomyces capitatus*, *Malassezia pachydermatis*, *Saccharomyces cerevisiae*, *Rhizopus oryzae*, *Lichtheimia corymbifera*, *Rhizomucor pusillus*, and *Apophysomyces elegans *are all reported to produce ECM in their biofilms [10, 11, 13, 163]. Therefore, ECM clearly plays a critical role in fungal biofilm resistance, particularly for *C. albicans*, from which we currently understand the greatest. It is one of the most significant and regulated resistance mechanisms utilized in the biofilm phenotype.

### 4.6. Persisters

Persister cells are an important mechanism of resistance in chronic infections [164], and a mechanism of resistance that has gathered some attention recently in fungal biofilms [165–167]. Persister cells are “dormant variants of regular cells that form stochastically in microbial populations and are highly tolerant to antibiotics” [168]. In *C. albicans* biofilms, a small subset of yeast cells have been described that are highly resistant to amphotericin B following adhesion, which is independent of upregulation of efflux pumps and cell membrane composition [76, 166]. The first study in fungi was to describe persister cells in fungi, described as a subpopulation of highly tolerant cells. In this study, *C. albicans* persisters were detected only in biofilms and not in different planktonic populations [166]. Reinoculation of cells that survived killing of the biofilm by amphotericin B produced a new biofilm with a new subpopulation of persisters, suggesting that these were not mutants but phenotypic variants of the wild type, and that attachment to a substratum initiated dormancy. The presence of persisters in *C. albicans*, *C. krusei*, and *C. parapsilosis* biofilms treated with amphotericin B was also described [169]. It was further hypothesized that the periodic application of antimicrobial agents may select for strains with increased levels of persister cells, so 150 isolates of *C. albicans* and *C. glabrata *were obtained from cancer patients who were at high risk for the development of oral candidiasis and who had been treated with topical chlorhexidine once a day. It was shown that the persister levels of the isolates varied from 0.2 to 9%, and strains isolated from patients with long-term carriage had high levels of persisters, whereas those from transient carriage did not [170]. Therefore, in this clinically relevant scenario, prolonged and ineffectual antifungal treatment may be beneficial to the biofilm population, which may be responsible for antimicrobial drug failure and relapsing infections.

The role of reactive oxygen species (ROS) in sessile *C. albicans* cells was investigated as they are known to be induced by high concentrations of miconazole, allowing 1-2% of miconazole-tolerant cells to persist [171]. Superoxide dismutases (Sods) were found to be differentially expressed by miconazole-treated sessile *C. albicans* cells compared to untreated cells. Inhibition of superoxide dismutase resulted in an 18-fold reduction of the miconazole-tolerant persister cells and increased endogenous ROS levels in these cells [165]. In biofilms from strains lacking Δsod4/Δsod5, at least 3-fold less miconazole-tolerant persisters were observed, and ROS levels were increased compared to the isogenic wild type. Therefore, miconazole-tolerant persisters are linked to the ROS-detoxifying activity of Sods. Whether this is the definitive molecular basis for *C. albicans *persister cells or a tolerance mechanism still remains to be determined, but these subpopulations are clearly another important fungal biofilm resistance mechanism.

### 4.7. Tolerance

Stress responses have become more fully realized as defined mechanisms of antifungal resistance. Pathogenic fungi encounter a range of physiological stresses from different environments, including temperature changes, ionic stress, changes in osmolarity, and oxidative stress, such as that experienced in the phagosomes of neutrophils [112]. These stresses are sensed through various receptors, which elicit responses through conserved signaling pathways. One of the most important is the mitogen-activated protein kinase (MAPK) signal transduction network, and the many others are subject to review [112]. It was first shown that the mitogen-activated protein kinase (MAPK) Mck1p, which is activated by contact stress, is involved in biofilm development. Moreover, the null mutant (*mck1*) biofilms were azole sensitive, in contrast to the sessile wild type and both planktonic strains. This indicates that Mck1p is involved in biofilm resistance through a stress pathway [172]. 

Calcineurin is a Ca^2+^-calmodulin-activated serine/threonine-specific protein phosphatase that plays many critical stress roles in the fungal cell, including amongst other things antifungal drug responses [173]. In planktonic cells, calcineurin is critical for *C. albicans *survival during azole treatment [174]. Inhibiting calcineurin pharmacologically or impairing calcineurin function genetically has synergistic activity with fluconazole and renders the azoles fungicidal against *C. albicans* [175]. Calcineurin has also been implicated in mediating resistance to the azoles in both *in vitro* and *in vivo* models of biofilm formation [176]. *C. albicans* cells in biofilms are up to 1,000-fold more resistant to fluconazole than planktonic cells, indicating that inhibitors could be used in combinations as novel therapeutic interventions to treat or prevent biofilms, whereas *C. dubliniensis *calcineurin inhibitors were unable to form biofilms [177]. Similar studies have evaluated the efficacy of a voriconazole-micafungin combination against *C. albicans *biofilms. Voriconazole significantly antagonized the fungicidal effect of micafungin against biofilms. To investigate the mechanism of antagonism, an inhibitor of calcineurin was evaluated, which reversed the voriconazole-induced resistance to micafungin [178]. This study also suggested that heat shock protein 90 (Hsp90) molecular chaperone played a role in this antagonism. Hsp90 regulates complex cellular circuitry in eukaryotes and potentiates the emergence and maintenance of resistance to azoles and echinocandins in *C. albicans*, at least in part via calcineurin [179]. It physically interacts with the catalytic subunit of calcineurin, keeping it stable and poised for activation [180]. A recent study led by the Cowen group demonstrated that genetic depletion of Hsp90 reduced *C. albicans* biofilm growth and maturation *in vitro* and interestingly impaired dispersal of biofilm cells [181]. It also abrogated resistance of *C. albicans* biofilms to the azoles, which was also shown *in vivo*. Furthermore, depletion of Hsp90 led to reduction of calcineurin and Mkc1 in planktonic but not biofilm conditions, suggesting that Hsp90 regulates drug resistance through different mechanisms. A marked decrease in matrix glucan levels was observed, providing a mechanism through which Hsp90 might regulate biofilm azole resistance. In *A. fumigatus*, pharmacological depletion of Hsp90 led to reduced resistance to the echinocandins [181]. Moreover, a recent investigation of the *C. glabrata *biofilm proteome demonstrated upregulation of a heat shock protein (Hsp12p) and other stress proteins (Trx1p, Pep4p) [182]. Therefore, targeting Hsp90 may provide a novel strategy for treating fungal biofilm infections.

## 5. Conclusions

Fungal biofilm resistance is multifaceted, involving some basic physical barriers and some complex regulatory processes. The evidence that has been collected over the past decade would suggest that as the biofilm changes from an adherent phenotype into a complex biofilm, then different mechanisms of resistance are utilised, that is, phase-specific mechanisms. Clearly, efflux pumps are utilised during the early to intermediate phases of biofilm development but are relinquished towards maturity as ECM is produced to “soak” and deplete antifungal agents. The density of the mature biofilm may act as a physical barrier, and reduction of growth rates under different environmental pressures can benefit the biofilm. Moreover, during hyphal growth, ergosterol biosynthesis is regulated by antifungal treatment, the direct and indirect targets of polyenes and azoles, respectively. Within the dense biofilms, where penetration of antifungal drugs is possible, persister cells phenotypes ensure survival, and in these stressed environments, global stress proteins kick into action to protect and maintain. Overall, the evidence highlights that fungal biofilm resistance is an inducible phenotype that is a part of a highly evolved series of molecular pathways that regulate biofilm development and homeostasis.
